# Increased Circulating Levels of Interleukin-6 Affect the Redox Balance in Skeletal Muscle

**DOI:** 10.1155/2019/3018584

**Published:** 2019-11-16

**Authors:** Laura Forcina, Carmen Miano, Bianca M. Scicchitano, Emanuele Rizzuto, Maria Grazia Berardinelli, Fabrizio De Benedetti, Laura Pelosi, Antonio Musarò

**Affiliations:** ^1^DAHFMO-Unit of Histology and Medical Embryology, Sapienza University of Rome, Laboratory Affiliated to Istituto Pasteur Italia-Fondazione Cenci Bolognetti, Via A. Scarpa 14, 00161 Rome, Italy; ^2^Istituto di Istologia ed Embriologia, Università Cattolica del Sacro Cuore, Fondazione Policlinico Universitario “Agostino Gemelli”, IRCCS, 00168 Roma, Italy; ^3^Department of Mechanical and Aerospace Engineering, Sapienza University of Rome, 00184 Rome, Italy; ^4^Division of Rheumatology and Immuno-Rheumatology Research Laboratories, Bambino Gesù Children's Hospital, 00146 Rome, Italy

## Abstract

The extent of oxidative stress and chronic inflammation are closely related events which coexist in a muscle environment under pathologic conditions. It has been generally accepted that the inflammatory cells, as well as myofibers, are sources of reactive species which are, in turn, able to amplify the activation of proinflammatory pathways. However, the precise mechanism underlining the physiopathologic interplay between ROS generation and inflammatory response has to be fully clarified. Thus, the identification of key molecular players in the interconnected pathogenic network between the two processes might help to design more specific therapeutic approaches for degenerative diseases. Here, we investigated whether elevated circulating levels of the proinflammatory cytokine Interleukin-6 (IL-6) are sufficient to perturb the physiologic redox balance in skeletal muscle, independently of tissue damage and inflammatory response. We observed that the overexpression of circulating IL-6 enhances the generation and accumulation of free radicals in the diaphragm muscle of adult NSE/IL-6 mice, by deregulating redox-associated molecular circuits and impinging the nuclear factor erythroid 2-related factor 2- (Nrf2-) mediated antioxidant response. Our findings are coherent with a model in which uncontrolled levels of IL-6 in the bloodstream can influence the local redox homeostasis, inducing the establishment of prooxidative conditions in skeletal muscle tissue.

## 1. Introduction

Basal levels of reactive oxygen and nitrogen species (ROS and RNS) in skeletal muscle operate as molecular signals and regulatory mediators of homeostatic processes, whereas the sustained production of free radicals is responsible for the oxidative damage of cellular components such as membrane lipids, proteins, and nucleic acids [1–4]. Thus, the impact of reactive molecules needs a tight modulation, guaranteed by the activation of a sophisticated system of antioxidant agents [5, 6]. A proper balance between prooxidant stimuli and antioxidant defence is necessary to allow the activation of redox-related physiologic pathways and to prevent the occurrence of oxidative alterations of muscle tissue [7–12]. One of the main sources of ROS production in skeletal muscle is the NADPH oxidase 2 (NOX2), an enzymatic complex responsible for the conversion of oxygen into superoxide, using NADPH as electron donor substrate [13–15]. As a compensatory mechanism, ROS can induce the nuclear translocation of Nrf2, which is considered the master regulator of the endogenous antioxidant defence [16, 17]. Indeed, Nrf2 protein regulates the expression of genes codifying for the main redox-regulating enzymes, such as glutamate-cysteine ligase (GCL), implicated in glutathione synthesis, NAD(P)H quinone dehydrogenase 1 (NQO1) and the heme-oxygenase 1 (HO-1), involved in superoxide detoxification, superoxide dismutase (SOD) engaged in ROS neutralization, and catalase (CAT), which can convert hydrogen peroxide (H_2_O_2_) into oxygen and water [18–24].

An efficient antioxidant chain can actively reduce the biodisponibility of superoxide, avoiding its combination with nitric oxide (NO) to generate RNS and thus favouring the physiologic signalling mediated by NO in skeletal muscle. Conversely, the uncontrolled production of ROS can induce an impairment of the Nrf2-dependent pathway, shunting muscle balance toward prooxidative conditions and leading to oxidative stress and tissue damage [25–28].

Indeed, the loss of the homeostatic redox balance represents a critical pathogenic mechanism contributing to muscle diseases [7–9, 11, 29]. In recent years, mounting evidence indicated that the excessive production of ROS plays a crucial role in a wide range of degenerative and inflammatory-related diseases, such as Duchenne muscular dystrophy (DMD), aging, diabetes, and cancer, leading to protein carbonylation and nitration, DNA damage, and RNA oxidation [1–4, 30, 31]. In particular, the perturbation of pivotal redox signalling pathways in dystrophic muscles has been related with the progression of DMD pathology and our studies supported the central role of ROS in promoting degenerative events in dystrophin-deficient muscles [27, 28, 31, 32]. However, the production of ROS has been generally considered as a secondary mechanism associated to chronic inflammation, which represents a major contributor to the pathogenesis of many musculoskeletal diseases [2, 33].

Although ROS production has been generally associated to the extent of chronic inflammation in DMD, we revealed an alteration of muscle redox status in presymptomatic DMD patients and in dystrophic animal models early before the onset of the pathology, characterized by myofiber necrosis and inflammatory infiltrate [27, 28]. Several studies support the existence of an interdependent relationship between inflammation and oxidative stress, and the interplay between these pathogenic mechanisms represents a critical issue to define the precise pathogenic events associated with neuromuscular diseases and to design more specific therapeutic approaches.

Among factors playing a critical role in skeletal muscle physiopathology and potentially linking inflammation and oxidative stress, Interleukin-6 (IL-6) is an elective candidate. IL-6 is a cytokine with pleiotropic functions in muscle environment, exerting positive and negative roles in tissue homeostasis. It has been extensively described that the dual nature of its action can be associated to the activation of different signalling pathways [34–36]. In particular, the activation of the classical signalling, mediated by the membrane IL-6 receptor alpha (mIL6R) and the ubiquitous receptor gp130, is physiologically restricted to responsive cells and has been involved in anti-inflammatory and proregenerative pathways in skeletal muscle [37–40]. The spectrum of action of IL-6 is known to be extended by the interaction of the cytokine with a soluble form of IL6R (sIL6R). This alternative receptor system activates the so-called trans-signalling with proinflammatory and profibrotic implications [41–44]. Of note, circulating levels of IL-6, which are normally undetectable, are increased in several disease conditions and IL-6 has been recognized as a proinflammatory, profibrotic, and prooxidant factor in different pathologic contexts [34, 35].

We recently provided evidence that IL-6 is causally linked to the pathogenesis of muscular dystrophy using two different experimental approaches: (i) increasing the circulating levels of IL-6 in mdx mice and analysing its effects in a stage normally spared by the absence of dystrophin and (ii) blocking IL-6 signalling in mdx mice at the prenecrotic stage and analysing its effects at the acute onset of the pathology. We demonstrated that the overexpression of IL-6 in dystrophic mdx mice (mdx/IL-6 murine model) induced the exacerbation of the dystrophic phenotype at 24 weeks of age, the stage in which the classical mdx model shows a stabilization of the disease [45], whereas the inhibition of IL-6 signalling in dystrophin-deficient mice reduces ROS accumulation, myonecrosis, and inflammation in the diaphragm muscle, which represents the most compromised muscular district in DMD [28, 36, 37, 46]. Noticeably, we recently highlighted a role for deregulated amounts of IL-6 in contributing to the alteration of local and systemic redox signalling markers in DMD patients and dystrophic mice [27, 28].

Nevertheless, it remained to be defined whether the alteration in redox balance was strictly related to the absence of dystrophin expression, and therefore to the disease, or whether increased levels of IL-6 are able alone to trigger a perturbation of redox balance in muscle milieu.

Thus, based on previous works performed on dystrophic murine models and human samples of DMD patients, we investigated the oxidative status of NSE/IL-6 muscle, in which the impact of increased levels of IL-6 cannot be biased against degenerating tissue and other related pathogenic mechanisms typical of dystrophic muscles, to better clarify the role of nonphysiologic levels of IL-6 in promoting the alteration of muscle oxidative balance. Indeed, although NSE/IL-6 mice have shown a decreased growth rate, no evidence of degeneration, fibrosis, or infiltrated inflammatory cells has been observed in the muscles of NSE/IL-6 mice.

We revealed that increased circulating levels of IL-6 act as a central player in deregulating the redox balance in diaphragm muscle. Of note, high plasma levels of IL-6 induce muscle atrophy and alteration in the functional performance of different muscle types, suggesting that, enhancing ROS production and impairing the antioxidant response, IL-6 fosters the molecular circuits generally associated to the extent of chronic tissue damage.

## 2. Materials and Methods

### 2.1. Mice

Wild-type C57Bl/6J mice and NSE/IL-6 transgenic mice [47] of 24 weeks of age were used in the current study. In particular, NSE/IL-6 mice, in which the rat neurospecific enolase (NSE) promoter drives the expression of human IL-6 cDNA, were derived from the original line 26 [46] and are characterized by elevated levels of circulating IL-6 since early after birth.

Animals were maintained as per the institutional guidelines of the animal facility of the unit of Histology and Medical Embryology. All the experiments on animal models were approved by the ethics committee of the Sapienza University of Rome-Unit of Histology and Medical Embryology and were performed in accordance with the current version of the Italian Law on the Protection of Animals.

### 2.2. RNA Extraction and Real-Time PCR Analysis

Liquid nitrogen-frozen diaphragm muscles of wild-type and NSE/IL-6 mice were powdered and homogenized in TRI Reagent (Sigma-Aldrich) by Tissue Lyser (Qiagen). Total RNA was extracted, and one microgram of each RNA sample was retrotranscribed using QuantiTect Reverse Transcription Kit (Qiagen), to obtain double-stranded cDNA. To synthesize single-stranded cDNA, ten nanograms of total RNA was reverse transcribed using the TaqMan MicroRNA Reverse Transcription Kit (Applied Biosystems). The analysis of mRNA and miRNA expression was performed on ABI PRISM 7500 SDS (Applied Biosystems). Specific TaqMan assays for SOD1, SOD2, NQO1, CAT1, Nrf2, GCL, SIRT1, Txnrd2, Atrogin1, MURF-1, CTSL, LC3, and miR-1 (Applied Biosystems) were used, and relative quantification was performed using Hprt and U6 snRNA as endogenous controls for mRNA and miRNA, respectively. Data were analysed using the 2-DDCt method and reported as mean fold change in gene expression relative to wild type.

### 2.3. Protein Extraction, Western Blot Analysis, and ELISA

Diaphragm muscles were isolated from 24-week-old wild-type and NSE/IL-6 mice, and liquid nitrogen powdered samples were homogenized in protein lysis buffer (Tris-HCl, pH 7.5/20 mM, EDTA/2 mM, EGTA/2 mM, Sucrose/250 mM, DTT/5 mM, Triton-X/0.1%, PMSF/1 mM, NaF/10 mM, SOV4/0.2 mM, Cocktail Protease Inhibitors/1x (Sigma-Aldrich)). For western blotting analysis, 70 *μ*g of protein extract was used, and filters were blotted with primary antibodies against gp91phox (BD Transduction), G6PD (Santa Cruz), Nitrotyrosine (Millipore), and GAPDH (Santa Cruz) and appropriate HRP-conjugated secondary antibodies (Bethyl Laboratories). Nitrotyrosine quantification was performed using the Stain-Free blot method for normalization (Criterion TGX Stain-Free Precast Gels; Bio-Rad).

Signals were captured by Chemi Doc™ XRS 2015 (Bio-Rad Laboratories), and densitometric analysis was performed using Image Lab software (version 5.2.1; Bio-Rad Laboratories©).

For the evaluation of circulating human IL-6, an ELISA was performed on serum samples using Quantikine® Colorimetric Sandwich ELISAs (R&D Systems), according to the manufacturer's protocol.

### 2.4. Histological Analysis and Dihydroethidium Staining

Diaphragm and EDL muscles from 24-week-old wild-type and NSE/IL-6 mice were embedded in tissue freezing medium and snap frozen in nitrogen-cooled isopentane. For general morphology, cryostat transversal sections were stained with Haematoxylin and Eosin according to standard protocols. Bright-field images were obtained using Axio Imager A2 microscope (Carl Zeiss Microimaging, Inc.) and analysed using ImageJ software (v.1.51j8; National Institutes of Health) to quantify the cross-sectional area of single myofibers. For dihydroethidium (DHE) staining, 10 *μ*m thick muscle cryosections were incubated with 5 *μ*M DHE (Molecular Probes #D23107) in PBS at 37°C for 30 minutes, photomicrographed using Axio Imager A2 microscope (Carl Zeiss Microimaging, Inc.), and processed by ZEN2 software (Blue edition). DHE-derived fluorescence was analysed by ImageJ software. The evaluation of the DHE-derived fluorescence was performed on at least five transversal sections of each muscle, and a minimum of eight fields arbitrarily chosen from each section was analysed.

### 2.5. Functional Analysis

Ex vivo functional analysis was performed on EDL muscles isolated from 24-week-old NSE/IL-6 and wild-type mice, as previously described [48]. Briefly, the muscle to be tested was mounted in a temperature-controlled chamber containing a Krebs-Ringer bicarbonate buffer continuously gassed with a mixture of 95% O_2_ and 5% CO_2_. Maximum force (Fmax) was evoked stimulating the muscle with 300 mA train of pulses delivered at 180 Hz. Specific force was computed as Fmax/CSA [48].

### 2.6. Data Analysis and Statistics

Statistical analysis was performed with GraphPad Prism Software (San Diego, CA, USA). All data were expressed as mean ± SEM. Differences among wild-type and NSE/IL-6 groups were assessed with Mann-Whitney test or Student's *t*-test assuming two-tailed distributions. *p* < 0.05 was considered statistically significant. The sample size was predetermined based on the variability observed in preliminary and similar experiments. All experiments requiring animal models were subjected to randomization based on litter.

## 3. Results

### 3.1. The Enhanced Expression of Circulating Interleukin-6 Promotes the Production and Accumulation of ROS in the Diaphragm Muscle

In this study, we extended previous works [27, 28] with the aim to define, in NSE/IL-6 transgenic mice, the contribution of circulating increased levels of IL-6 on redox balance perturbation. NSE/IL-6 mice express human IL-6 (hIL-6) cDNA under the rat neurospecific enolase (NSE) promoter, resulting in increased amounts of circulating IL-6 early after birth [47]. To verify whether hIL-6 levels were maintained elevated in the bloodstream during adulthood, serum levels of transgenic IL-6 were measured, by ELISA, in 24-week-old NSE/IL-6 mice. The protein content of hIL-6 was of 13.8 ± 2.8 ng/ml, compared to the undetectable levels of the wild-type mice (data not shown). Noticeably, the circulating levels of IL-6 observed in the serum of NSE/IL-6 mice were comparable with the levels reported for the serum of mdx/IL-6 mice at the same age [45].

**Figure 1:**
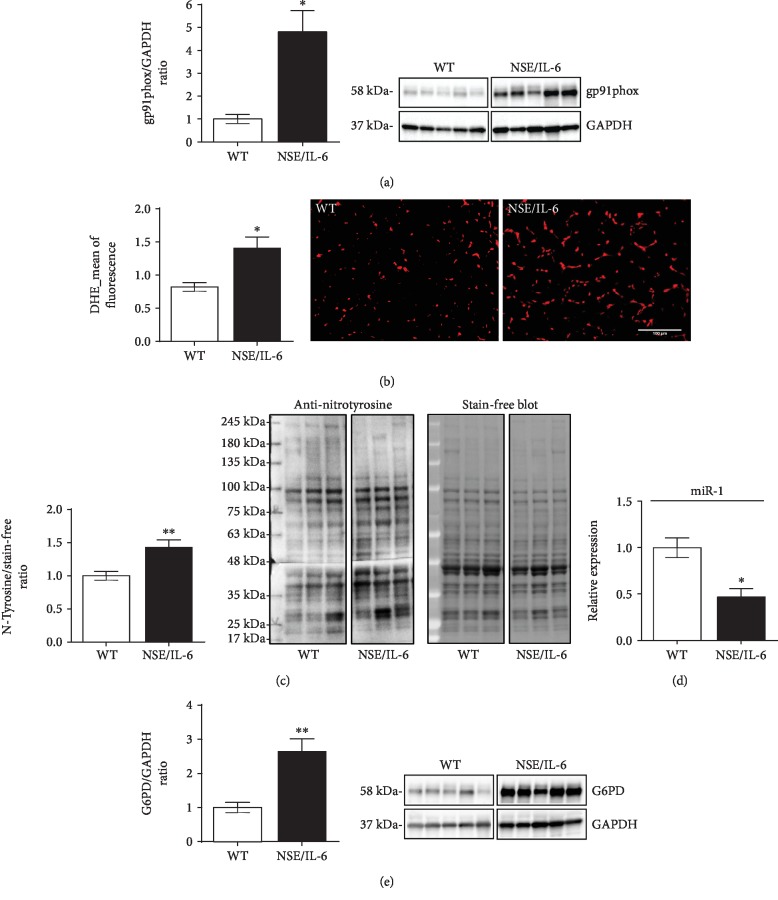
IL-6 induced the enhanced ROS production and accumulation in the diaphragm muscle. Western blot analysis (right panels show representative images) for the expression of gp91phox (a) and G6PD (e) proteins in 24-week-old NSE/IL-6 and wild-type (WT) mice. Values represent mean ± SEM; *n* = 5 to 6 mice per group. ^∗^*p* < 0.05, ^∗∗^*p* < 0.005 by Student's two-tailed unpaired *t*-test. Lanes were run on the same gel but were not contiguous. Original images are shown in [Supplementary-material supplementary-material-1] and [Supplementary-material supplementary-material-1]. (b) Quantitative analysis (left panel) of the mean intensity of fluorescence derived from DHE staining on diaphragm muscle sections from 24-week-old NSE/IL-6 and WT mice. Right panels show representative images of DHE staining on muscle sections of indicated genotypes (scale bar 100 *μ*m). Values represent mean ± SEM; *n* = 3 to 5 mice per group. ^∗^*p* < 0.05 by unpaired *t*-test. (c) Western blot analysis for the detection of nitrated proteins (right panels show representative images) in the diaphragm muscle of NSE/IL-6 and WT mice. Values represent mean ± SEM; *n* = 6 mice per group. ^∗∗^*p* < 0.005 by unpaired *t*-test. Lanes were run on the same gel but were not contiguous. Original images are shown in [Supplementary-material supplementary-material-1]. (d) Real-time PCR analysis of the expression of miR-1 performed on diaphragm muscle samples from NSE/IL-6 and WT mice at 24 weeks of age. Values represent mean ± SEM; *n* = 3 to 5 mice per genotype. ^∗^*p* < 0.05 by unpaired *t*-test. DHE: dihydroethidium; G6PD: glucose 6-phosphate dehydrogenase.

We thus analysed the generation, accumulation, and impact of oxidizing products in the diaphragm muscle of both 24-week-old NSE/IL-6 and wild-type (WT) mice (Figure 1). The rationale of considering the diaphragm muscle of 24-week-old NSE/IL-6 mice lies with the evidence that this specific muscle showed a significant redox alteration in age-matched dystrophic mice expressing comparable IL-6 levels of NSE/IL-6 transgenic mice [45].

We evaluated the expression of gp91phox protein, the catalytic subunit of the enzymatic complex NADPH oxidase 2 (NOX2), responsible for the conversion of molecular oxygen to superoxide (O_2_^−^) [13, 15]. A significant increase of gp91phox protein expression was observed in NSE/IL-6 muscle compared to that in wild-type mice (Figure 1(a)), suggesting an enhanced generation of superoxide in muscles exposed to nonphysiologic levels of circulating IL-6.

Since superoxide can be efficiently neutralized by the endogenous antioxidant defence, we verified whether the enhanced expression of gp91phox, observed in NSE/IL-6 mice, could effectively result in increased production and persistence of reactive radicals in muscle tissue. The accumulation of ROS in skeletal muscle has been evaluated by DHE, a fluorescent redox-sensitive probe able to mainly detect not only superoxide anion but also peroxynitrite (ONOO^−^) and hydroxyl (^·^OH) radicals [49–54]. Quantitative analysis performed by fluorescence microscopy revealed that the diaphragm muscles of NSE/IL-6 mice presented a significant increase of DHE-derived fluorescence compared to wild-type muscles (Figure 1(b)), indicating that IL-6 overexpression induces the accumulation of ROS within muscle tissue.

It is well known that overproduced ROS can interact with nitric oxide (NO), a molecular mediator of critical cellular functions, giving rise to peroxynitrite (ONOO^−^) [55]; peroxynitrite can, in turn, interact with proteins leading to the nitration of tyrosine residues [56].

To investigate whether the abundance of ROS detected in NSE/IL-6 diaphragm muscle can induce the oxidative modification of proteins, we evaluated the presence of nitrotyrosine in NSE/IL-6 and wild-type muscles as a marker of nitrooxidative stress (Figure 1(c)). We revealed that the content of nitrated proteins was higher in NSE/IL-6 diaphragm compared to wild-type muscle, indicating that NOX2-derived ROS can be combined with NO, with consequent induction of nitrooxidative damage. Notably, protein nitration not only represents a posttranslational modification able to alter protein structure and function but can also disturb the physiologic NO signalling [56–59].

The NOX2-dependent production of O_2_^−^ is strictly dependent on the availability of nicotinamide adenine dinucleotide phosphate (NADPH), although this substrate is also necessary for the activity of antioxidant systems contributing to the neutralization of ROS. The cytosolic enzyme designated to the maintenance of cellular levels of NADPH is the glucose-6-phosphate dehydrogenase (G6PD), which appears as a common player in both pro- and antioxidant systems [60]. The altered expression of G6PD and of its negative regulator miR-1 has been described in dystrophic muscles, as a direct consequence of the impaired NO signalling [57]. Thus, we investigated whether the enhancement of prooxidant stimuli in muscle milieu induced by IL-6 can be responsible for the alteration of redox-connected molecular circuits, such as the miR-1/G6PD axis (Figures 1(d) and 1(e)) [57, 61]. We observed a significant downregulation of miR-1 (Figure 1(d)) and a significant increase of G6PD protein (Figure 1(e)) in the diaphragm muscles of 24-week-old NSE/IL-6 mice compared to wild-type mice. These data indicated that the overexpression of IL-6 can induce, through the deregulation of redox-related circuits, a sustained production of NADPH which can in turn foster the NOX2-dependent ROS production.

### 3.2. The Nrf2-Mediated Antioxidant Response Is Impaired in NSE/IL-6 Diaphragm Muscle

The oxidative impact of ROS in skeletal muscle is physiologically counterbalanced by the activation of the endogenous antioxidant response [2, 62]. A central mediator in this protective mechanism is the redox-sensitive transcription factor Nrf2 [19, 63]. Under prooxidant conditions, Nrf2 is known to act as a master regulator of the antioxidant program, by inducing the expression of genes involved in the neutralization of reactive species such as superoxide dismutase (SOD1/2), thioredoxin reductase (Txnrd2), catalase (CAT-1), and NAD(P)H quinone dehydrogenase (NQO1). In addition, Nrf2 can influence the efficiency of the glutathione system modulating the expression of genes involved in the glutathione synthesis, namely, glutathione cysteine ligase (GCL) [64, 65].

**Figure 2:**
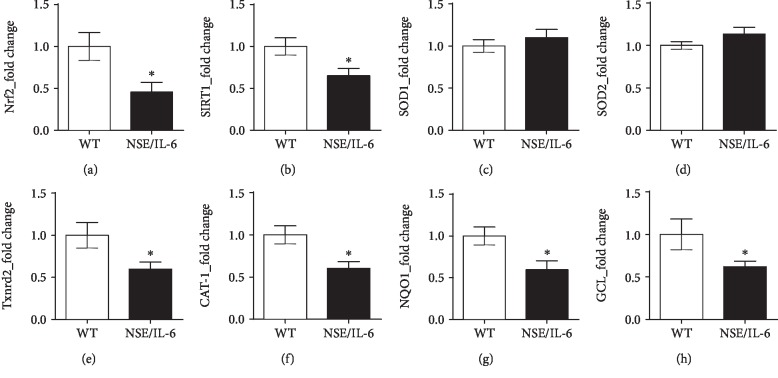
Nrf2-dependent antioxidant genes are differentially modulated in NSE/IL-6 diaphragm muscles. Real-time PCR analysis of the expression of Nrf2 (a), SIRT1 (b), and Nrf2-dependent genes: SOD1 (c), SOD2 (d), Txnrd2 (e), CAT-1 (f), NQO1 (g), and GCL (h). Gene expression analysis was performed on diaphragm muscle samples deriving from wild-type (WT) and NSE/IL-6 mice at 24 weeks of age. Values represent mean ± SEM; *n* = at least 4 mice per genotype. ^∗^*p* < 0.05 by unpaired *t*-test. Nrf2: nuclear factor erythroid 2-related factor 2; SIRT1: sirtuin 1; SOD: superoxide dismutase; Txnrd2: thioredoxin reductase 2; CAT-1: catalase-1; NQO1: NAD(P)H quinone dehydrogenase 1; GCL: glutamate-cysteine ligase.

We previously reported that the imbalanced redox status of dystrophic muscles involved both the enhanced production of ROS and the impinged antioxidant response. In fact, it has been proposed that elevated levels of ROS within muscle tissue might overcome the endogenous antioxidant system [28]. To define whether sustained levels of circulating IL-6 might be sufficient, independently from a genetic degenerative disease, to determine an alteration of the local antioxidant profile of skeletal muscle, we analysed important mediators of the antioxidant response in diaphragm muscles of both adult mice overexpressing IL-6 and wild-type control mice (Figure 2). We found that the expression of Nrf2 (Figure 2(a)) and SIRT1 (Figure 2(b)), pivotal players of the redox-signalling cascade [66], was downmodulated in 24-week-old NSE/IL-6 diaphragm compared to wild-type muscle. Nevertheless, gene expression analysis revealed a differential modulation of the Nrf2 target genes (Figures 2(c)–2(h)). Indeed, we observed a significant downregulation of CAT-1 (Figure 2(f)), NQO1 (Figure 2(g)), and GCL (Figure 2(h)) in NSE/IL-6 diaphragm compared to wild type, whereas SOD1 (Figure 2(c)) was expressed at similar levels in both NSE/IL-6 and wild-type muscles. Notably, the reduced expression of GCL and NQO1 can impair the availability of reduced glutathione (GSH), contributing to the undeterred accumulation of ROS.

Since mitochondria are known to be active producers of ROS, as well as sensor of the cellular redox status [67], we further analysed the expression of important antioxidant genes involved in the mitochondrial redox regulation, namely, SOD2 and Txnrd2 (Figures 2(d) and 2(e)). Although SOD2 expression was not modulated (Figure 2(d)), we found a significant reduction of Txnrd2 expression in NSE/IL-6 diaphragm muscles compared to wild type (Figure 2(e)). Of note, Txnrd2 is a thioredoxin reductase enzyme participating in the maintenance of a proper mitochondrial redox balance and the impairment of Txnrd2 gene expression has been found in aged muscles and has been associated to the loss of mitochondrial integrity in cardiomyocytes [68, 69]. These data suggest that the deregulation of Nrf2-dependent genes can influence the mitochondrial scavenging of ROS in muscles deriving from IL-6-overexpressing mice.

### 3.3. Increased Systemic Levels of IL-6 Impinge Morphofunctional Properties of Skeletal Muscle

The uncontrolled generation and accumulation of reactive molecules and the impairment of antioxidant systems are, as a whole, a critical stressor contributing to the disturbance of muscle function [70]. Indeed, oxidative stress has been recognized as a pivotal mechanism contributing to skeletal muscle alteration, inducing the oxidative modification of cellular components and participating to the imbalance between protein synthesis and degradation [71].

**Figure 3:**
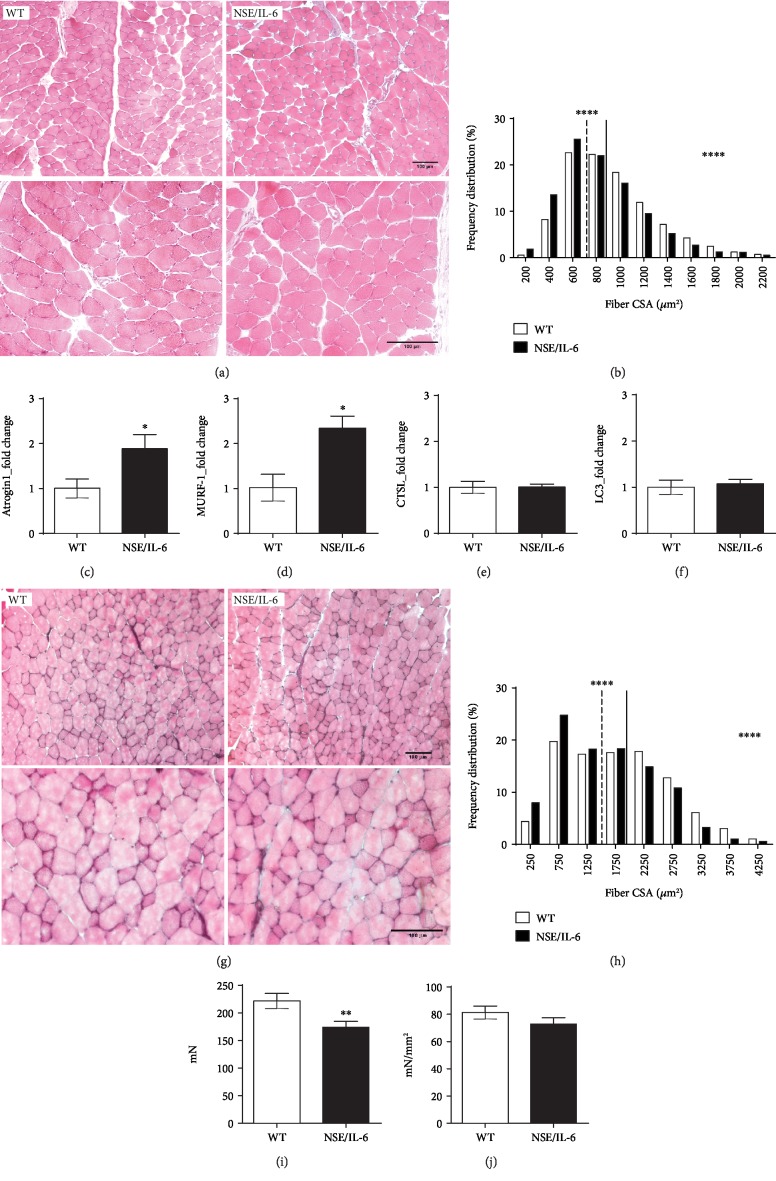
Morphofunctional analysis of NSE/IL-6 and wild-type muscles at 24 weeks of age. (a) Representative image of Haematoxylin and Eosin staining of transverse sections of diaphragm from 24-week-old wild-type (WT) and NSE/IL-6 mice. Scale bar, 100 *μ*m. (b) Frequency distribution of myofiber cross-sectional area (CSA) in transgenic (NSE/IL-6) and wild-type (WT) diaphragm muscle. Data are represented as medians; *n* = 4; ^∗∗∗∗^*p* < 0.0001. Real-time PCR analysis for the expression of Atrogin1 (c), MURF-1 (d), CTSL (e), and LC3 (f). Values represent mean ± SEM; *n* ≥ 5 mice per genotype. ^∗^*p* < 0.05 by unpaired *t*-test. (g) Haematoxylin and Eosin staining of transverse section of extensor digitorum longus (EDL) muscles from indicated genotypes. Scale bar, 100 *μ*m. (h) Frequency distribution of myofiber cross-sectional area (CSA) in transgenic (NSE/IL-6) and wild-type (WT) EDL muscle. Data are represented as medians; *n* ≥ 3; ^∗∗∗∗^*p* < 0.0001. Physiological properties of EDL muscles from 24-week-old wild-type (WT) and NSE/IL-6 mice: (i) Maximum force (mN) and (j) specific force (mN/mm^2^). Data are represented as mean ± SD of EDL muscle maximum force (i) and specific force (j); *n* > 31; ^∗∗^*p* < 0.01. Atrogin1: MAFbx/Atrogin1; MURF-1: Muscle RING Finger-1; CTSL: cathepsin L; LC3: microtubule-associated protein 1 light chain 3.

To evaluate the effective impact of elevated levels of ROS on skeletal muscle tissue, we performed histological analysis of the diaphragm muscle of 24-week-old NSE/IL-6 and wild-type mice. Noticeably, the evaluation of the general morphology of the NSE/IL-6 diaphragm muscle did not reveal evident signs of infiltrating mononuclear cells, degenerating fibers, or the presence of regenerating centrally nucleated myofibers (Figure 3(a)). However, morphometric analysis, performed on the diaphragm muscle of NSE/IL-6 and wild-type mice, revealed a significant reduction in the cross-sectional area (CSA) of single myofibers in NSE/IL-6 mice compared to wild-type mice (Figure 3(b)). The reduced calibre of fibers observed in transgenic muscles suggested that elevated levels of IL-6, along with the increased amount of intracellular ROS, might induce muscle atrophy in NSE/IL-6 mice.

Indeed, it is well known that IL-6 is a catabolic factor able to induce muscle atrophy when its levels are deregulated [72] and a role for ROS in promoting proteolysis in skeletal muscle has been proposed [71, 73]. We thus analysed pivotal mediators of the main protein degradation pathways involved in muscle atrophy [74]. In particular, we evaluated the expression markers of the ubiquitin-proteasome pathway, the muscle-specific E3 ubiquitin ligases MAFbx/Atrogin1 and Muscle RING Finger-1 (MURF-1) (Figures 3(c) and 3(d)). Real-time PCR analysis revealed a significant upregulation of both Atrogin1 (Figure 3(c)) and MURF-1 (Figure 3(d)) expression in the diaphragm muscle of 24-week-old NSE/IL-6 mice compared to age-matched wild type. In contrast, the analysis of important players of the autophagy-lysosome system, cathepsin L (CTSL) and the microtubule-associated protein 1 light chain 3 (LC3), did not reveal significant variation in gene expression between NSE/IL-6 and wild-type muscles (Figures 3(e) and 3(f)).

In order to evaluate the impact of nonphysiologic amounts of circulating IL-6 levels on a fast-twitch muscle, morphofunctional analysis was performed on extensor digitorum longus (EDL) muscle of both NSE/IL-6 and wild-type mice at 24 weeks of age (Figures 3(g)–3(j)). As reported for the diaphragm muscle (Figure 3(b)), also the EDL muscle showed a reduced cross-sectional area of single myofibers when compared to the EDL of age-matched wild-type mice (Figure 3(h)). In addition, functional analysis showed a significant reduction (-22%) in the capability of NSE/IL-6 EDL to generate maximum force (Figure 3(i)). No significant differences were measured in the specific force (Figure 3(j)).

Overall, these data support the role of deregulated levels of circulating IL-6 in affecting muscle mass with a significant impact on muscle function.

## 4. Discussion

Reactive oxygen species are physiologically produced, especially in metabolic active tissues like skeletal muscle, and are considered important secondary mediators involved in homeostatic processes. However, the excessive production and accumulation of reactive oxygen and nitrogen species are known to induce cell damage by the peroxidation of membrane lipids, protein carbonylation/nitration, and genome insults [1–4]. The extent of ROS production and oxidative damage has been related to the pathogenesis of chronic inflammatory conditions and degenerative diseases such as Duchenne muscular dystrophy [8, 31, 32]. Although inflammation and ROS production have been described as interrelated processes, the precise mechanism linking inflammation and oxidative stress has still to be defined. We recently reported that the overexpression of the proinflammatory cytokine IL-6 in dystrophin-deficient mice (mdx/IL-6 model) is able to exacerbate the dystrophic muscle phenotype, fostering pathologic changes in skeletal muscle and closely approximating the disease progression of DMD patients [27, 28, 31]. Since the increased expression of circulating IL-6 in dystrophic mice has been related to the perturbation of muscle redox signalling even before the occurrence of early signs of the pathology, namely, necrosis and inflammation, we hypothesized that elevated levels of IL-6 can play a pivotal role in the alteration of the muscle redox balance, triggering pathogenic mechanisms associated with muscle degeneration.

Here, we investigated the direct impact of IL-6 on the homeostatic muscle redox balance in a noninjured context, verifying whether high levels of IL-6 are sufficient to induce the establishment of a prooxidant environment in skeletal muscle. To address this issue, we analysed the diaphragm muscle of adult NSE/IL-6 mice characterized by systemically elevated levels of IL-6 [47].

A central mediator of ROS production in skeletal muscle tissue is gp91phox protein, the catalytic subunit of NOX2 complex, which is known to be overexpressed in muscles of DMD patients and dystrophic mice [13, 15, 27, 28, 75]. Thus, we evaluated the extent of prooxidative stimuli in muscles exposed to elevated levels of circulating IL-6 to verify whether nonphysiologic levels of IL-6 can induce the overproduction and accumulation of ROS in a nondystrophic background. We observed not only a strong upregulation of gp91phox protein in the diaphragm muscle of adult NSE/IL-6 mice, compared to age-matched wild type, but also an increased accumulation of ROS in NSE/IL-6 muscles, highlighted by the extent of DHE-derived fluorescence. These data indicate that the overexpression of IL-6, independently from the absence of a functional dystrophin protein, is able to induce the establishment of prooxidant conditions in skeletal muscle tissue that can result in the occurrence of oxidative stress.

The detrimental impact of ROS on cellular and molecular processes was supported by the increased content of nitrated proteins, which are considered markers of nitrooxidative damage and have been related to the alteration of the physiologic NO signalling [3].

It is well known that NADPH content represents a critical factor supporting the activity of both NOX2 and glutathione system, one of the main cellular antioxidant systems. Indeed, NADPH is the electron carrier necessary for the conversion of molecular oxygen into superoxide, by NOX2, and for the reconstitution of reduced/active glutathione. The enzyme responsible for the production of NADPH is G6PD, a member of the NO/HDAC2/miR-1 molecular circuit, which is known to be altered in DMD muscles and reported as directly deregulated by the absence of dystrophin protein [57]. In particular, it has been described that in dystrophin-deficient muscles, the reduced availability of nitric oxide resulted in the decreased S-nitrosylation of proteins, including epigenetic regulators as HDAC2. Nonnitrosylated HDAC2 actively reduces the expression of the muscle-specific microRNA, miR-1, leading to the enhanced expression of G6PD, with a consequent increase of NADPH content in dystrophic muscles. This deregulated pathway provides substrate mainly for the activity of NOX2, since the glutathione system is known to be impaired in DMD muscles. Thus, to investigate potential mechanisms associated to a redox disequilibrium and responsible for the enhanced production of ROS in skeletal muscle exposed to elevated levels of serum IL-6, we evaluated central mediators of the HDAC2/miR-1/G6PD axis in NSE/IL-6 diaphragm muscles. We revealed a significant downmodulation of miR-1 and a concomitant upregulation of G6PD expression in NSE/IL-6 diaphragm compared to wild-type muscle. These data indicate that increasing levels of IL-6 not only induced the enhanced expression of NOX2 protein but are also involved in the perturbation of critical redox-related molecular circuits which are able to potentiate the NOX2-dependent ROS production in skeletal muscle.

Another critical mechanism participating in the maintenance of a proper cellular redox balance is the activation of the endogenous antioxidant response. It has been described that Nrf2, a redox-sensible transcription factor, can induce the activation of the endogenous antioxidant defence and the expression of important players involved in the cellular stress response, such as IL-6 [19–23]. Indeed, it has been reported that not only Nrf2 can induce IL-6 expression by interacting with a responsive element in the IL-6 promoter but also that IL-6 can in turn enhance the Nrf2-mediated antioxidant response [76]. However, under pathologic conditions such as sepsis, characterized by a massive cytokine release, including IL-6, the antioxidant defence is impaired suggesting an hormetic stress response to IL-6 cytokine [77]. We recently reported that elevated levels of IL-6 in dystrophic muscles are related to the impairment of the Nrf2-mediated antioxidant response, promoting the extent of local and systemic oxidative damage and contributing to the progression of the pathology [27, 28]. Thus, to clarify the impact of abnormal levels of IL-6 on the physiologic response to oxidative conditions, we evaluated the Nrf2-dependent antioxidant response in NSE/IL-6 and wild-type muscles. The reduced expression of Nrf2 and the deregulation of Nrf2-dependent antioxidant genes, observed in NSE/IL-6 mice, clearly indicate that IL-6 can impair the detoxification of ROS in muscle tissue. Indeed, although the expression of SOD1 gene in NSE/IL-6 muscles was similar to wild-type levels, we revealed an impaired expression of CAT-1, NQO1, and GCL genes in mice overexpressing IL-6. In particular, CAT-1 is responsible for the neutralization of H_2_O_2_, NQO1 is a NADPH-dependent superoxide scavenger, and GCL represents the rate-limiting enzyme for the de novo synthesis of glutathione (GSH) [78, 79]. Thus, our data indicate that in NSE/IL-6 muscles the superoxide produced by NOX2 can be dismutated into H_2_O_2_, whereas the detoxification of hydrogen peroxide can be impaired. In addition, the reduced expression of NQO1 and GCL in NSE/IL-6 diaphragm suggests that the G6PD-derived NADPH can be preferentially used as a substrate for ROS production instead of ROS neutralization. The alteration of the muscle redox status in IL-6-overexpressing mice is further supported by the reduced expression of SIRT1, a multifunctional regulator of the cellular antioxidant defence, which has been also correlated to the modulation of inflammatory factors and to the biogenesis of mitochondria [80].

Mitochondria are pivotal cell source of ROS deriving from unpaired electrons produced during the oxidative phosphorylation process [81]. Based on their active production of oxidant species, these organelles retain the ability to efficiently counteract the oxidative impact of free radicals, maintaining their structural and functional integrity, through the action of antioxidant effectors such as catalase, MnSOD, and the glutathione and the thioredoxin systems. Of note, SOD2 and Txnrd2 genes have been recognized as potential target of Nrf2 [82–84]. Thus, we investigated whether the impaired expression of Nrf2 in NSE/IL-6 muscle could affect the expression of important regulators of the mitochondrial redox status. We found a significant downmodulation of Txnrd2, but not SOD2 gene expression, in adult NSE/IL-6 muscles compared to control mice. These results suggest that the alteration of the muscle redox status induced by high levels of IL-6 might negatively influence the efficacy of the mitochondrial antioxidant defence, with possible implication on mitochondrial integrity.

The dysfunction of cellular organelles, along with the altered structure of membranes and proteins, is a feature of a deregulated redox condition, namely, oxidative stress, which can profoundly affect the morphofunctional properties of skeletal muscle [70]. Since oxidative stress is known to result from the imbalance between ROS generation and neutralization, we investigated whether the altered muscle redox balance, observed in our model, might effectively afflict muscle phenotype.

Although the histological evaluation of the NSE/IL-6 diaphragm muscle did not reveal evident signs of necrosis, fibrosis, and mononuclear infiltrating cells, morphometric analysis showed a significant reduction of myofiber calibre in NSE/IL-6 diaphragm compared to wild-type muscle. We thus reasoned that the reduced size of single myofibers in muscles exposed to aberrant levels of systemic IL-6 might be related to the activation of atrophic pathways, considering the acknowledged catabolic action of both IL-6 and ROS in skeletal muscle. Molecular analysis was performed to evaluate markers of the main atrophy-related molecular networks, and we revealed a significant upregulation of mediators of the ubiquitin-proteasome pathway in the diaphragm muscle of 24-week-old NSE/IL-6 mice compared to age-matched control mice, whereas the expression markers of the autophagic system were not modulated. These data supported the hypothesis of a direct involvement of IL-6 in the induction of a ROS-mediated alteration of skeletal muscle tissue.

In addition, morphofunctional analysis performed on EDL muscles of both NSE/IL-6 and wild-type mice revealed that also the EDL muscle, which has been recognized as a fast-twitch muscle, showed a reduced cross-sectional area of single myofibers when compared to age-matched wild-type mice. Since skeletal muscle atrophy is known to be characterized by the loss of both muscle mass and contractile properties [70], we evaluated the ability of EDL muscles to generate force upon electrical stimulation. The EDL muscle derived from 24-week-old NSE/IL-6 mice revealed a significant reduction of the tetanic force compared to age-matched wild type, thereby confirming the induction of an atrophic phenotype.

The observed decline of important morphofunctional parameters in NSE/IL-6 muscle suggested that elevated levels of systemic IL-6 might exert a local action, through the enhanced generation and accumulation of ROS, negatively influencing skeletal muscle tissue and function.

## 5. Conclusions

**Figure 4:**
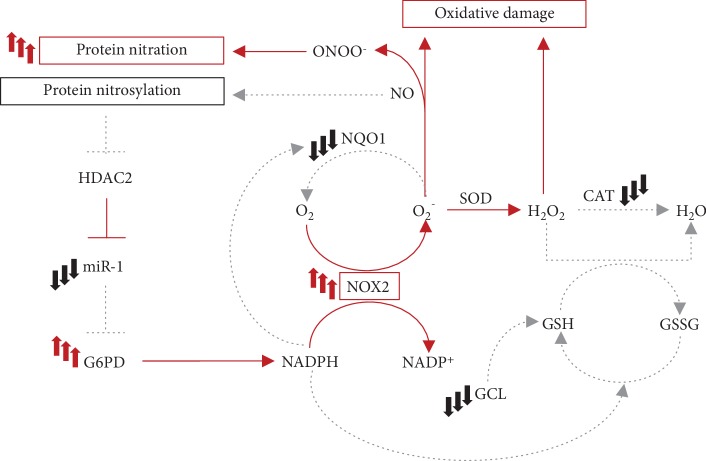
A proposed model of the impact of elevated levels of circulating IL-6 on skeletal muscle redox balance. The reported scheme represents molecular circuits involved in the generation and neutralization of reactive species in skeletal muscle. Red lines indicate mechanisms that are potentially enhanced by elevated levels of circulating IL-6. Grey dot lines represent processes which might be impaired in NSE/IL-6 muscle. In the presence of nonphysiologic amounts of serum IL-6, NOX2 expression is enhanced in diaphragm muscle, inducing a sustained generation of superoxide (O_2_^−^). The downmodulation of NQO1 in muscle tissue exposed to increased levels of serum IL-6 indicates that the NOX2-derived superoxide might be not efficiently neutralized. On the other hand, O_2_^−^ is converted by SOD into hydrogen peroxide (H_2_O_2_), whilst its further detoxification can be impaired by the reduced expression of CAT. H_2_O_2_ can also be neutralized through the oxidation of glutathione. The reduced expression of the rate-limiting enzyme to produce glutathione (GSH), GCL, might reflect an impaired activity of the glutathione system. The excess of O_2_^−^ can also interact with nitric oxide (NO) inducing protein modifications. Moreover, the altered regulation of the NO signalling pathway might induce a deregulated expression of glucose 6-phosphate dehydrogenase (G6PD), the enzyme responsible for the production of NADPH, further enhancing the activity of the NOX2 complex in a feed-forward circuit. NOX2: NADPH oxidase 2; NQO1: NAD(P)H quinone dehydrogenase 1; SOD: superoxide dismutase; CAT: catalase; GCL: glutamate-cysteine ligase; ONOO^−^: peroxynitrite; HDAC2: histone deacetylase 2; GSSG: oxidized glutathione.

IL-6 has been recognized as a myokine exerting important functions in skeletal muscle physiopathology. Based on the opposite roles of IL-6 in body and muscle homeostasis, the spectrum of action of IL-6 is tightly regulated and its production is physiologically maintained at very low levels in serum. Elevated circulating levels of IL-6 are instead associated to different pathologic conditions in human and murine models. Herein, we take the advantage of the NSE/IL-6 mouse model to evaluate the impact of altered serum levels of IL-6 on skeletal muscle physiology. We highlighted that circulating IL-6, when deregulated, profoundly affects muscle homeostasis inducing the establishment of prooxidant conditions, which influence the local redox balance that can in turn amplify tissue degeneration under pathologic conditions (Figure 4).

## Data Availability

The datasets used to support the findings of this study are included in the present article and are available from the corresponding author upon request.

## Supplementary Materials

Supplementary Materials Figure S1 the original image of western blot of gp91phox and GAPDH proteins. Figure S2: original images of western blot for nitrotyrosinated proteins and of stain-free blot. Figure S3: the original image of western blot of G6PD and GAPDH proteins. We refer to these figures in Section 3.1.
