# Supplementary material for: Increased Circulating Levels of Interleukin-6 Affect the Redox Balance in Skeletal Muscle
Source: Oxid Med Cell Longev. 2019 Nov 16;2019:3018584. doi: 10.1155/2019/3018584 (PMC6881749; doi:10.1155/2019/3018584)
Supplement: Supplementary Materials — Figure S1 the original image of western blot of gp91phox and GAPDH proteins. Figure S2: original images of western blot for nitrotyrosinated proteins and of stain-free blot. Figure S3: the original image of western blot of G6PD and GAPDH proteins. We refer to these figures in Section 3.1. [file 3018584.f1.pdf]

Supplementary Materials

Increased circulating levels of Interleukin-6 affect the redox balance in skeletal muscle

Laura Forcina, Carmen Miano, Bianca M. Scicchitano, Emanuele Rizzuto, Maria Grazia Berardinelli, Fabrizio De Benedetti, Laura Pelosi, and Antonio Musarò

Correspondence: Antonio Musarò; DAHFMO-Unit of Histology and Medical Embryology, Sapienza University of Rome. Via A. Scarpa, 14 Rome 00161, Italy.

E-mail: antonio.musaro@uniroma1.it

Supplementary Figure 1.

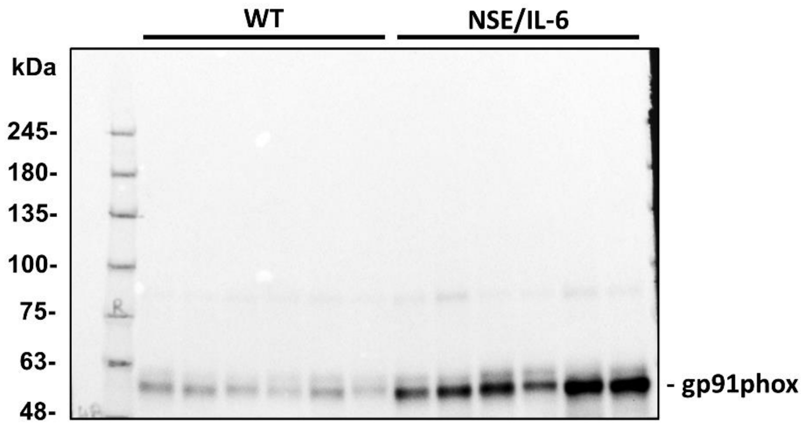

(a)

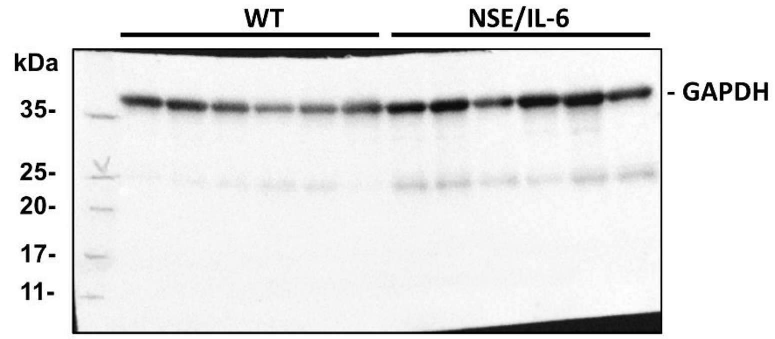

(b)

Figure S1. Western Blot analysis of gp91phox (a) and GAPDH (b) proteins in wild type (WT) and NSE/IL-6 transgenic mice. Cropped bands reported as representative images are shown in Figure 1(a).

Supplementary Figure 2.

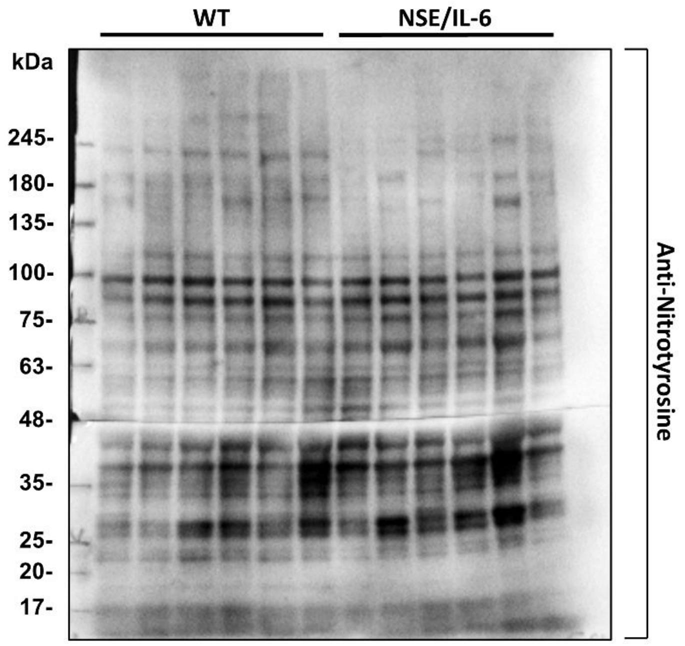

(a)

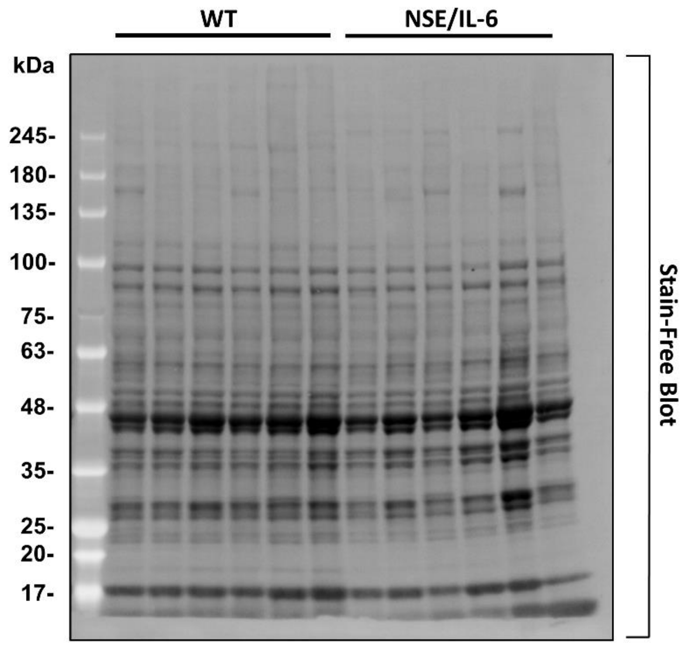

(b)

Figure S2. Western Blot analysis of nitrotyrosinated proteins (a) in wild type (WT) and NSE/IL-6 transgenic mice. Stain Free blot image (b) was used as control for protein loading.
Selected lanes, reported as representative images, are shown in Figure 1(c).

Supplementary Figure 3.

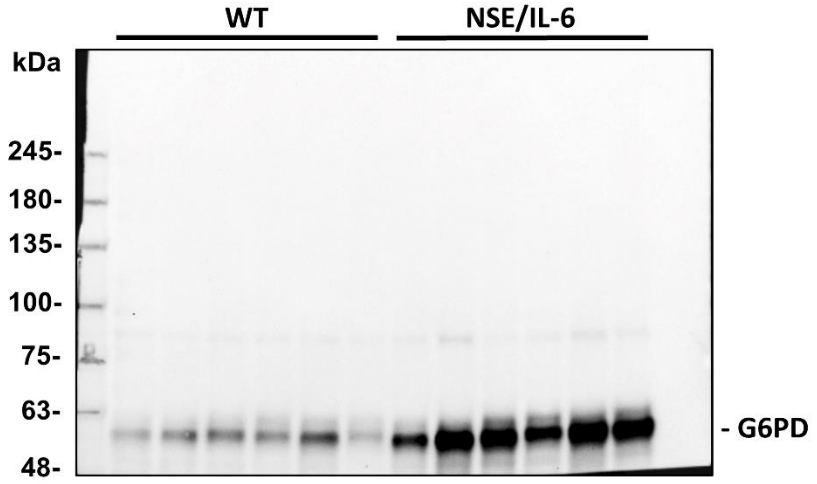

(a)

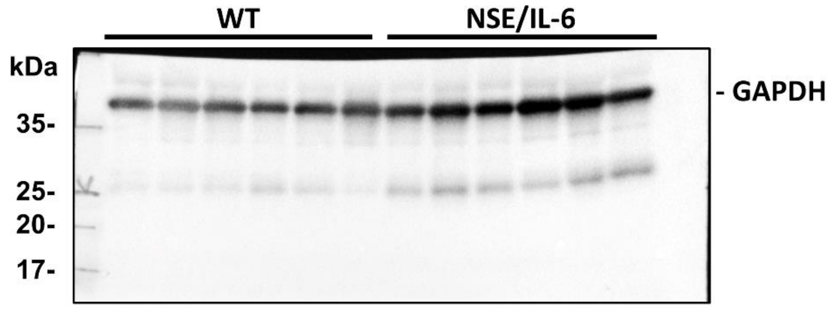

(b)

Figure S3. Western Blot analysis of G6PD (a) and GAPDH (b) proteins in wild type (WT) and NSE/IL-6 transgenic mice. Cropped bands reported as representative images are shown in Figure 1(e).
